# Hypoxia Tolerance in Teleosts: Implications of Cardiac Nitrosative Signals

**DOI:** 10.3389/fphys.2018.00366

**Published:** 2018-04-12

**Authors:** Alfonsina Gattuso, Filippo Garofalo, Maria C. Cerra, Sandra Imbrogno

**Affiliations:** Department of Biology, Ecology and Earth Sciences, University of Calabria, Rende, Italy

**Keywords:** heart, hypoxia, nitric oxide, nitrite, teleosts

## Abstract

Changes in environmental oxygen (O_2_) are naturally occurring phenomena which ectotherms have to face on. Many species exhibit a striking capacity to survive and remain active for long periods under hypoxia, even tolerating anoxia. Some fundamental adaptations contribute to this capacity: metabolic suppression, tolerance of pH and ionic unbalance, avoidance and/or repair of free-radical-induced cell injury during reoxygenation. A remarkable feature of these species is their ability to preserve a normal cardiovascular performance during hypoxia/anoxia to match peripheral (tissue pO_2_) requirements. In this review, we will refer to paradigms of hypoxia- and anoxia-tolerant teleost fish to illustrate cardiac physiological strategies that, by involving nitric oxide and its metabolites, play a critical role in the adaptive responses to O_2_ limitation. The information here reported may contribute to clarify the molecular and cellular mechanisms underlying heart vulnerability vs. resistance in relation to O_2_ availability.

## Introduction

Molecular oxygen (O_2_) is essential for life. A limited O_2_ supply leads to tissue hypoxia which often results in organ damage.

When compared to terrestrial animals, water-breathing organisms are more likely to be exposed to wider temporal and spatial variations of O_2_ supply. This is largely due to the inherent properties of the water and to the rapid fluctuations in the pattern of O_2_ production and consumption (Nikinmaa et al., 2011). Several animal species are adapted to tolerate regular and often severe hypoxia. This is the case of various fish, frog, and turtle species that tolerate anoxia, and some snakes and insects that can endure severe hypoxia (Hermes-Lima and Zenteno-Savín, 2002). Teleost fish exhibit a very large spectrum of O_2_ sensitivity, moving from species showing an extraordinary ability to tolerate hypoxia and anoxia to species that dramatically suffer O_2_ deprivation. Among teleost fish, examples of hypoxia/anoxia resistance are the members of the cyprinid genus Carassius, such as the goldfish (*Carassius auratus*), and the crucian carp (*Carassius carassius*), which exhibit a striking capacity to survive and remain active for long periods under low O_2_, even tolerating anoxia (Bickler and Buck, 2007). This capacity is correlated with the ability to generate ethanol as anaerobic end-product, which is acid–base neutral, in contrast with the normal glycolytic end-product lactic acid. Among cyprinids, the zebrafish (*Danio rerio*) is characterized by a growth-dependent transition from hypoxia tolerance to sensitivity (Padilla and Roth, 2001). Its genome is fully sequenced and this is of benefit for studying the components of hypoxia-resistance pathways in fish.

Despite the different abilities of vertebrates to tolerate a limited O_2_ availability in the environment or in internal tissues, many studies in mammalian and non-mammalian models (see Fago and Jensen, 2015, for references) suggest a common set of concerted physiological responses. They basically include depression of O_2_ consumption rates, protection against oxidative damage, and, at least in air-breathing species, redistribution of blood flow into the circulation. All these responses require the activation of a complex network of intracellular cascades, such as those related to nitric oxide (NO) and its metabolites, nitrite and nitrate, that represent common signaling molecules able to control and coordinate the molecular circuits that sustain adaptive hypoxia-dependent physiological responses (Fago and Jensen, 2015).

The heart is a major target of hypoxia. As largely documented in mammals, O_2_ deprivation is accompanied by changes in cardiac muscle metabolism, reactivation of fetal gene programs and hypertrophy responses, modifications of the extracellular matrix composition, mitochondrial biogenesis and arrangement, as well as of the expression of intracellular effectors [i.e., the NO pathway, hypoxamiRs, Hypoxia Inducible Factor (HIF), etc.] (Fago and Jensen, 2015). A large number of studies on mammals have recognized the critical role of NO and its metabolites, in particular nitrite and S-nitrosothiols (SNO; formed when Cys thiols react with NO^+^), in the mechanisms which control the cardiac response to low O_2_ availability. This role has been recently extended to non-mammalian vertebrates, including fish, in which a growing body of evidence has documented the cardioprotective role of NO and its related nitrosative signals, under hypoxic challenges.

In this review we will illustrate the cardiac role of the nitric oxide synthase (NOS)/NO/nitrite system with emphasis to hypoxia-activated cardio-protective effects of nitrite, a major source of NO under low O_2_. We will mainly provide information on the cardiac nitrosative signaling of teleost species characterized by a high ability to tolerate hypoxia/anoxia. This feature makes them valuable models to whom deserve a renewed attention to explore the mechanisms that contribute to survive low O_2_, also in a translational perspective for human cardioprotection. For the reader who is unfamiliar with the adaptive physiology of fish, we will outline their phenotypic plasticity in relation to anoxia and hypoxia, and to the different mechanisms that allow O_2_ detection. In the subsequent adaptive response, the heart, as a major effector, is modulated to properly sustain organism requirements.

## Environmental stress response in vertebrates

Many vertebrate species are able to face a wide range of environmental changes in abiotic parameters that include O_2_ availability, temperature, pH, and salinity. A common universal strategy characterizes the homeostatic response to different environmental stresses. This strategy requires important modifications from molecular to organismal level. An example is the response to hypoxia/anoxia that, as in case of the thermal stress, is characterized by a decrement in bioenergetic demand/production whose consequences are energy conservation, osmotic balance, and substrate economy (Boutilier, 2001 and references therein). The universal nature of the stress response is confirmed by genomic and post-genomic studies showing that the phenotypic flexibility of many species in response to hypoxia (as well as to hypothermia) involves the same pattern of genes that influence ATP and protein turnover, energy conservation, and stress factors release (Hochachka and Somero, 2002).

Two physiological adaptations confer the ability to cope with environmental stresses: “capacity adaptations” (i.e., the condition in which organisms preserve normal levels of both activity and homeostasis, enabling them “to grow and reproduce under the harsh conditions”) and “resistance adaptations” (i.e., the condition in which organisms enhance their resistance even losing homeostasis, enabling them “to avoid or survive the stress until conditions become favorable again”) (Cossins and Bowler, 1987). The reaction to stressors varies between individual members of a given species and represents a combination of factors, i.e., the appraisal of the environmental change and the ability to cope (Broom and Johnson, 1993; Koolhaas et al., 1999). As stated by Wilson et al. (1994), phenotypes within one species have a differential fitness and this differentiation represents adaptive individual differences in resource use and response to risk.

The different responses to stress can be clustered in two different phenotypes within the same species: proactive and reactive (Wilson et al., 1994). Proactive animals are characterized by high sympathetic and locomotor activity, while reactive individuals show low mobility and sympathetic activity (Van Raaij et al., 1996). Proactive individuals are usually audacious and more aggressive with respect to their reactive counterparts; these different activity levels between the two phenotypes have been observed both in wild (Montiglio et al., 2010) and laboratory environments (Tran and Gerlai, 2013). As observed in the teleost rainbow trout (*Oncorhynchus mykiss*) exposed to hypoxia, the proactive phenotype corresponds to an escaping and “non-surviving fish,” showing a strong avoidance behavior with a consequent high energy expenditure and instantaneous beginning of anaerobic metabolism. In contrast, the reactive fish remains quiet and survives thanks to the delaying activation of anaerobic mechanisms (Schjolden et al., 2005 and references therein).

Another trait of the adaptive response to low O_2_ is its relationship with animal development and growth. In general, it can be assumed that adults are less hypoxia-tolerant than neonatal and embryonic vertebrates (Crowder et al., 1998). If exposure to hypoxia occurs during juvenile or adult life, the effect is reversible. In contrast, if the stress takes place during the development, its influence persists throughout the life (Padilla and Roth, 2001).

## Anoxia and hypoxia tolerance of the fish heart

Fish, as well as amphibians and reptiles, are characterized by an impressive ability to survive long period of partial and/or complete O_2_ deprivation. Three major adaptations allow these animals to face anoxia: deep metabolic depression, tolerance of acidosis and osmotic stress, prevention and/or restoration of cell damage induced by the huge radical production during re-oxygenation (see for review Driedzic and Gesser, 1994; Bickler and Buck, 2007). Moreover, long-term survival to anoxic stress requires massive accumulations of glycogen in critical tissues, and an extreme metabolic depression in specific body districts, and this allows the extension of anoxia tolerance to the whole organism (Hochachka, 1986).

Fish show a very high variety of phenotypes characterized by different abilities to cope with O_2_ fluctuations. At the basis of this plasticity is the expression of specific genes. Many investigations have recently attempted to identify these genes, and this resulted in the development of a database of Fish Hypoxia Responsive Genes (HRGFish) which currently covers 818 gene sequences and 35 genes (including HIF, myoglobin, and glucose transporters) from 38 fishes (Rashid et al., 2017).

The formidable adaptations shown by teleost fish to face low and very low O_2_ availability is typically related to the anaerobic capacity of the animal. This is exemplified by an early study by Mathur (1967) on the Indian cyprinid *Rasbora daniconius* showing that, if placed in a hermetically sealed glass jar, this teleost survives for more than 100 days. This resistance to protracted anoxia is supported by a notable anaerobic potential and the ability to escape from acidosis caused by the anaerobic waste-product increase (e.g., lactate).

Because of their very high resistance to minimum rates of water O_2_ saturation and a wide range of temperatures (from <4°C up to >38°C), Cyprinids, such as the common carp *Cyprinus carpio*, and its related specie, the crucian carp *C. carassius*, are largely recognized as major experimental models in which analyze morpho-functional adaptation to environmental or laboratory hypoxia. In these species, a significant metabolic down-regulation (down to 30%), which allows glycogen storing, is fundamental to surviving anoxia (Nilsson, 2001; Lutz and Nilsson, 2004).

**Figure 1:**
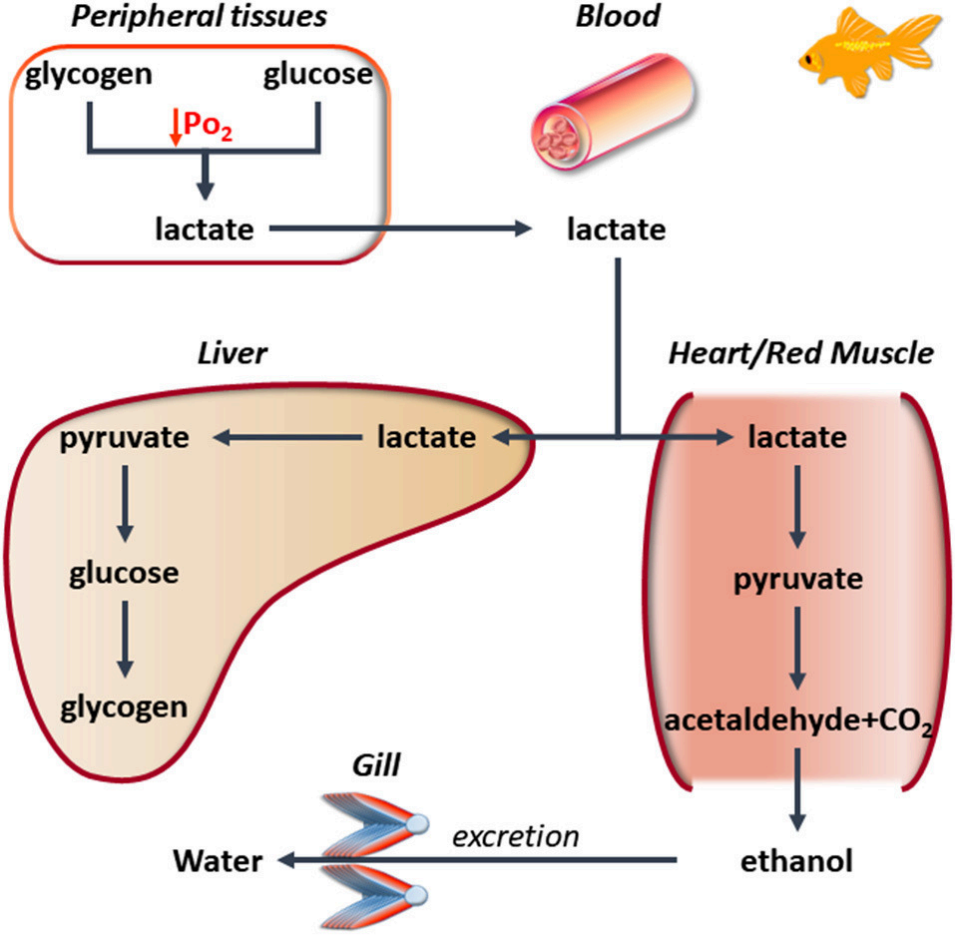
A simplified scheme illustrating the metabolic cooperation among different tissues in hypoxia-tolerant Cyprinid in the presence of low O_2_. Under these conditions, the muscle is able to activate the conversion of lactate (also produced by other organs) to ethanol. This is excreted by the gills, thus preventing lactate accumulation.

**Figure 2:**
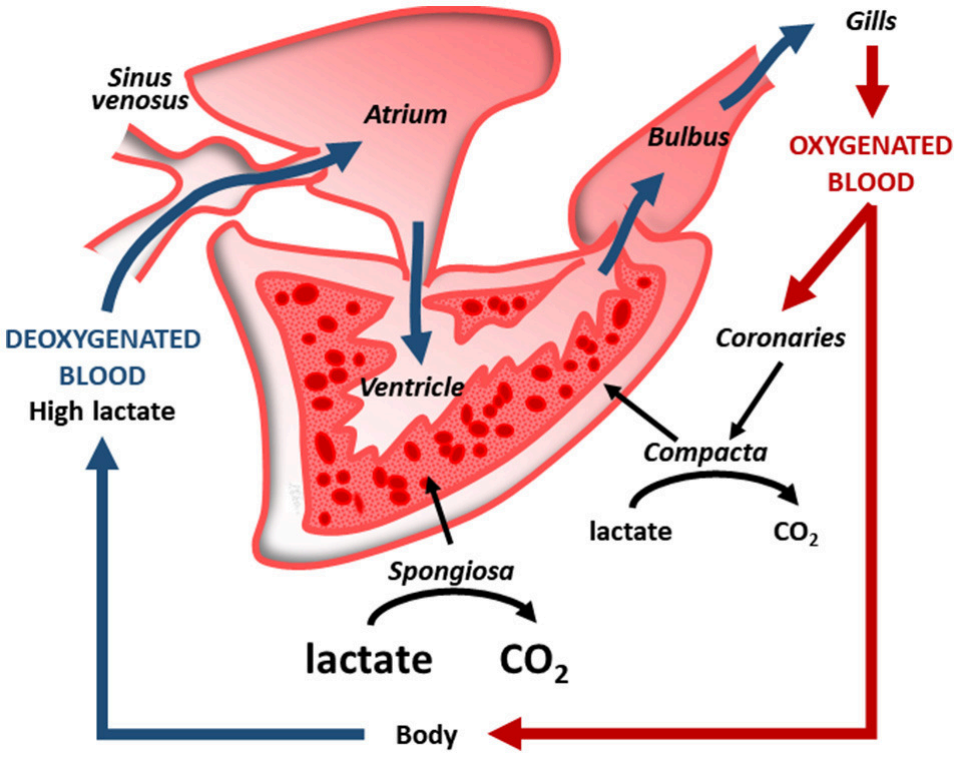
Intracardiac cooperation in a coronarized teleost heart (e.g., tuna). With respect to the compact layer, supplied with coronary oxygenated blood, the spongy layer, supplied by venous blood, has a greater capability to oxidize lactate to CO_2_ (modified from Tota et al., 2011 with permission).

Information on these fascinating models of hypoxia/anoxia resistance mainly comes from studies aimed to explore the mechanisms of defense of their hearts. Remarkable cardiac interspecific differences have emerged. In fact, while in the common carp, a critical decrease in heart function occurs during 24 h of severe hypoxia, the crucian carp conserves normal cardiac activity and autonomic cardiovascular control up to 5 days of anoxia at 8°C (Stecyk et al., 2004). This preserved cardiovascular function allows the crucian carp to effectively perfuse with blood both gills and liver. The appropriate perfusion mobilizes huge amount of glucose from the large hepatic glycogen store to all tissues, allowing, at the same time, lactate transport to the muscle where it is converted to ethanol (Nilsson, 2001). This, being less harmful than lactate, prevents acidosis. Additionally, the easily diffusible ethanol is quickly removed by the branchial epithelium thanks to the very effective blood perfusion. Accordingly, a preserved heart performance is the basis for improving anoxia resistance of the whole piscine organism thanks to the improved metabolic and functional cooperation among single organs (Figure 1). In this context, also the cooperation between different cardiac regions contributes to the response to low O_2_. In the cardiac ventricle of the bluefin tuna (*Thunnus thynnus*), the subendocardial trabeculae (*spongiosa*), essentially perfused by venous blood, are capable to metabolize lactate more than the compact subepicardial myocardium, which is completely perfused by oxygenated blood through the coronary vessels coming from the gills (Figure 2); this allows the *spongiosa* to face a reduced O_2_ availability (reviewed in Tota et al., 2011).

The role of fuel substrates, in relation to the response to low O_2_ availability, has been also analyzed. Studies on the isolated and perfused eel heart show that glucose supply is essential for maintaining the cardiac performance during acute anoxic stress (see references in Imbrogno, 2013). In *Anguilla rostrata*, characterized by a marked anoxic endurance, if oxidative phosphorylation is blocked by perfusion with NaCN, endogenous glycogen stores are consumed regardless of the presence or absence of glucose in the medium (Bailey et al., 2000). Of note, down-regulation of oxidative phosphorylation is critical for heart endurance under protracted anoxia. As shown in the *Carassius* species, a strong metabolic depression is essential for the conservation of glycogen stores allowing fish to resist very long period of anoxia (Bickler and Buck, 2007; Vornanen and Haverinen, 2016).

During anoxia exposure, anoxia tolerant hearts show an *in vivo* cardiac ATP demand lower than their cardiac maximum glycolytic potential (i.e., the maximum ATP production solely from glycolysis) (Farrell and Stecyk, 2007). Two strategies, for achieving this have been proposed: a naturally low routine cardiac ATP demand that can be sustained by anaerobic glycolysis (as in the case of crucian carp, *C. carassius*), or a considerably down-regulation of cardiac ATP demand to a level that can be sustained by glycolytic ATP production (e.g., in *C. carpio)*. In the case of the *C. carpio*, hypoxic bradycardia may represent a strategy to protect the heart since it allows the depression of cardiac power output and thus ATP requirement, reducing the need of oxygen for several hours (Farrell, 2007). In the case of *C. carassius*, during anoxia, anaerobic ATP production is sufficient to power the heart to pump, thus it is not necessary to activate bradycardia (Stecyk et al., 2004).

## Oxygen-sensing and hypoxia/anoxia responses

Detection of environmental, hematic, or tissue O_2_ is a crucial task of the homeostatic response of vertebrates to hypoxia/anoxia. Complex and integrated systems and effectors are present in the different classes, phyla, and species, and even within the same species. A critical step is the activation of sensors which detect O_2_ during the initial, acute phase of hypoxia. As a consequence, catecholamines are secreted and the cardiovascular activity is modulated so that vascular tissue perfusion is adjusted to balance O_2_ supply and demand (Buckler, 2007; Milsom and Burleson, 2007). If the hypoxia persists, these mechanisms activate long-term responses that require the regulation of gene expression [i.e., the hypoxia inducible factor (HIF) transcription factor family] and this sustains and potentiates the initial response.

Detection of environmental and blood hypoxia relies on the activation of extremely specialized chemoreceptors. They are characterized by a very high sensitivity to small changes in PO_2_ in either the external environment, or the internal micro environment, depending on their position. They are strategically located in regions exposed to O_2_ fluctuations, as in the case of the neuroepithelial cells (NECs) of the gills, the neuroepithelial bodies of the airways, and the carotid bodies in the vasculature.

In fish, regardless their phyletic position, a major role in O_2_-sensing is attributed to branchial NECs whose location and orientation is not uniform across species. They can be distributed either across the gill arches, or outside the gills in the oro-branchial cavity (see Milsom, 2012 for references; Gilmour and Perry, 2007; Milsom and Burleson, 2007). In addition, NECs may be oriented externally or internally or both, and this provides the animals with a selective ability to detect PO_2_ variations in water, and blood, or in both environments (reviewed by Milsom, 2012). It is generally assumed that hypoxia-tolerant species responds primarily to arterial hypoxaemia, while less tolerant fish respond more immediately to aquatic hypoxia (Sundin et al., 2000).

Externally-oriented chemoreceptors represent a primitive O_2_ sensing mechanism that disappeared early in vertebrate evolution, since they are absent in the obligate air-breathing South American lungfish, *Lepidosiren paradoxa* (Sanchez et al., 2001). In this fish, only blood hypoxia, but not water hypoxia, induces a rapid ventilator response, suggesting a prevalent role of internal rather than of branchial receptors.

Branchial NECs are differently sensitive to low PO_2_ and this correlates with the degree of hypoxia tolerance/intolerance of the various species. For example, in the hypoxia tolerant goldfish, isolated NECs do not respond to low PO_2_ until severe hypoxic or even anoxic levels. An increased ventilation is observed only if goldfish are exposed at water PO_2_ that are lower (25 mmHg: Tzaneva et al., 2011) than those (110 mmHg) eliciting the ventilatory response in the hypoxia sensitive zebrafish (Vulesevic et al., 2006). This may be advantageous during prolonged environmental anoxia to avoid continuous stimulation of stimulation of chemoreceptors without the possibility of increasing O_2_ uptake.

An interesting aspect of fish O_2_-sensitivity is the relationship with development. As observed in zebrafish, animals may change from hypoxia/anoxia tolerance to hypoxia sensitivity during growth. The zebrafish embryo is initially anoxia-tolerant but becomes hypoxia-sensitive between 2 and 3 days post-fertilization (dpf), when it begins to show hyperventilation under hypoxia (Padilla and Roth, 2001; Mendelsohn et al., 2008). This suggests that the chemoreflex induced by hypoxia appears before gill NECs are fully functional, and is presumably mediated by extrabranchial chemoreceptors (Jonz et al., 2015).

## Three hypotheses for oxygen-sensing and downstream responses

Studies in mammals have proposed three hypotheses for O_2_ sensing: a “membrane hypothesis,” a “mitochondrial/metabolic hypothesis,” and a more recent recent “gasotransmitter hypothesis” (reviewed by Prabhakar and Peers, 2014; Dzal et al., 2015; López-Barneo et al., 2016). Once activated, all these mechanisms converge on K^+^ currents of chemoreceptors cells with consequent neurotransmitter release and activation of the physiological modulation of ventilation and perfusion to maintain homeostasis.

### Membrane hypothesis

In mammals, chemoreceptor-dependent O_2_ sensing relies on the modulation of plasmalemmal K^+^ channels. Different types of K^+^ channels are involved, including background K^+^ (K_B_) channels (Buckler, 1999), large conductance Ca^2+^-activated K^+^ currents (K_Ca_) (Peers, 1990; Wyatt and Peers, 1995), TASK-like background K^+^ channels (Buckler, 2007), and kv3 and kv4 channels (Sanchez et al., 2002; Pérez-García et al., 2004). Modulation of these channels regulates membrane permeability and cell excitability, according to a common scheme for chemotransduction. Inhibition of the resting K^+^ current allows membrane depolarization, opening of voltage-dependent Ca^2+^ channels, neurotransmitter secretion and excitation of afferent neurons to cardio-respiratory centers (Nurse, 2005).

Available data suggest that also in fish, hypoxic chemotransduction involves O_2_-sensitive K^+^ currents, as supported by evidence in gill NECs of zebrafish (Jonz et al., 2004; Qin et al., 2010) and channel catfish (*Ictalurus punctatus*) (Burleson et al., 2006). While in zebrafish, the cell current appears to be mediated by background K^+^ (K_B_) channels (Jonz et al., 2004; Qin et al., 2010), similar to TASK-like background K^+^ channels of the mammalian carotid body (Buckler, 2007), in catfish the hypoxic response seems to be mediated by O_2_-sensitive voltage-dependent outward K^+^ (K_v_) current (Burleson et al., 2006).

Variations in membrane Ca^2+^ fluxes and intracellular Ca^2+^ concentrations, with consequent stimulation of neurotransmitter release, are presumably also involved in fish O_2_ chemoreception (Jonz, 2014). However, while in both zebrafish and catfish, gill NECs express a small number of Ca^2+^-activated K^+^ channels (K_Ca_) (Jonz et al., 2004; Burleson et al., 2006; Qin et al., 2010), the goldfish gill NECs express predominantly K_Ca_ channels, with a minor contribution of K_B_ and Kv (Zachar and Jonz, 2012) and L-type Ca^2+^ channels (Zachar et al., 2017). These data suggest a species-specific expression of ion channels that, modulated as a consequence of PO_2_ changes, participate to O_2_ sensing.

### Mitochondrial/metabolic hypothesis

Under hypoxia, the mitochondrial ATP generation is reduced and this affects many intracellular effectors, and thus a large spectrum of cell functions. Two major cellular events are proposed downstream the hypoxia-dependent ATP decrease. One is the inhibition of K^+^ TASK-like background channels. This may result in membrane depolarization and initiation of the electric activity and of voltage-dependent Ca^2+^ entry (Varas et al., 2007). The second is the increased cytosolic AMP/ATP ratio followed by the activation of AMP-activated protein kinase (AMPK). This, by inhibiting O_2_ sensitive K^+^ channels (K_B_ and K_Ca_) leads to chemoreceptors depolarization (Evans et al., 2005, 2009; Wyatt and Evans, 2007; Wyatt et al., 2007). In fish, no data are available concerning the involvement of mitochondria in O_2_ sensing. Only few data suggest a role for AMPK in the downstream response to anoxia of tolerant species. As observed in heart and brain of the tolerant crucian carp, AMPK induces metabolic depression and ethanol secretion only under anoxia, but not under hypoxia (Stensløkken et al., 2008), consistent with a quiescent kinase until complete anoxia is achieved (Pamenter, 2014). This may be an advantage for hypoxia-tolerant species since may allow to increase the depth and/or duration of hypoxia they can tolerate before the activation of AMPK-mediated metabolic adjustments becomes a necessity. In this way, protein synthesis, and other AMPK downregulated anabolic pathways, continue to function under hypoxia along with the ability to preferentially shunt blood flow to at-risk organs (e.g., brain and heart) (Pamenter, 2014).

### Gasotransmitters hypothesis

NO, Carbon Monoxide (CO), and Hydrogen sulfide (H_2_S), are mediators of O_2_ sensing in chemoreceptors. As shown in the mammalian carotid body, under normoxia, NO and CO are inhibitory, while H_2_S is excitatory. Regardless the type of response, their effects occur via ion channels modulation: NO causes glomus cell hyperpolarization by inhibiting Ca^2+^ channels and activating K^+^ channels; CO also activates these latter channels, preventing cell depolarization, while H_2_S inhibit them (for review see Prabhakar and Peers, 2014). Under hypoxia, both NO and CO production decrease, as their enzymatic activity requires molecular O_2_, leading to a reduced inhibition of L-type Ca^2+^ channels and to the closure of K^+^ channels. In contrast, H_2_S generation increases under hypoxia. This condition is associated with the inhibition of maxiK and TASK-like channels (see Prabhakar and Peers, 2014). All the above effects result in membrane potential depolarization, neurotransmitter release, and afferent neurons activation.

In fish, few data suggest a role for NO and CO in O_2_ chemoreception and in the control of breathing, while more robust information indicates that H_2_S is directly involved in O_2_ sensing and in the hypoxic response (Olson et al., 2008).

A NO-dependent O_2_-sensing mechanism has been proposed based on the identification of nNOS in neuroepithelial cells of adult and larvae of the zebrafish (Porteus et al., 2015). According to Perry and Tzaneva (2016), like in mammalian glomus cells (Summers et al., 1999), NO may inhibit ion channels involved in neurosecretion. This is supported by the NO-dependent regulation of intracellular Ca^2+^ observed in the melanophore of the Indian snakehead *Channa punctatus* (Biswas et al., 2011) and in the growth hormone release of goldfish (Chang et al., 2014). Apart from its putative involvement in O_2_ chemoreception, in teleosts, as well as in mammals, NO may play a role in the hypoxia-elicited response via a modulation of the ventilatory performance. This is supported by evidence in zebrafish that NO produced by nNOS expressed in branchial NECs, modulates the response to hypoxic stimuli by inhibiting and stimulating ventilation in adult and larvae, respectively (Porteus et al., 2015). As hereafter discussed, NO and its metabolites play a crucial role in the responses to O_2_ limitation, representing signaling molecules able to control and coordinate the molecular circuits that sustain adaptive hypoxia-dependent physiological responses.

In fish, as in mammals, O_2_ sensing involves the HO-dependent generation of CO. As observed in the goldfish, HO-1 (the hypoxia inducible isoform) is present in branchial NECs and its inhibitory effect on ventilation in goldfish acclimated to 7°C may reflect the HO-1-mediated production of CO within NECs (Tzaneva and Perry, 2014). The involvement of CO in the control of the ventilatory response has been also proposed by data in zebrafish, in which HO-1 expression in both larvae (skin NECs) and adult (gill NECs), is indicative of an endogenous CO production (Tzaneva and Perry, 2016). Once produced in branchial peripheral chemoreceptors, CO may affect the piscine ventilatory response via a modulation of intracellular Ca^2+^ and neurotransmitter release (Tzaneva and Perry, 2014), as in mammalian glomus cells (Overholt and Prabhakar, 1997; Prabhakar, 1999). This view is supported by the presence of both L-type Ca^2+^ and K_Ca_ channels in gill NECs of the goldfish which respond to hypoxia by increasing intracellular Ca^2+^ and synaptic vesicle activity (Zachar et al., 2017).

Several evidence in mammals indicate that H_2_S acts as an O_2_ sensor (Olson, 2011) and a mediator of hypoxic signaling (Peng et al., 2010). H_2_S generation, by constitutive cystathionine γ-lyase (CSE), cystathionine β-synthase (CBS), and 3-mercaptopyruvate sulfurtransferase (3-MST)/cysteine aminotransferase (CAT) (Kimura, 2011), is linked to O_2_ availability. In fact, it is present at low levels under normoxia, because of its oxidation by the mitochondrial electron transport chain enzymes, but increases under hypoxia, when the activity of the mitochondrial electron transport chain is reduced (Olson, 2011). In the carotid body, the mechanism involves a reduced activity of HO-2 which in turn increases CSE activity and thus H_2_S production; however, the role of CSE and H_2_S in the hypoxia sensing is not universally accepted. Recently, Wang et al. (2017) reported that in glomus cells from CSE^−/−^ mice, hypoxia-dependent effects on TASK-like channels, intracellular calcium, and ventilation were not modified. Even if the study does not provide a role for CSE in acute hypoxia sensing, this cannot be excluded (Wang et al., 2017), also considering that CSE inhibition affects the hypoxia response in chronic pathological states (Yuan et al., 2016).

H_2_S plays a role in O_2_ sensing also in fish. The first evidence was obtained in rainbow trout and zebrafish where, under hypoxia, the gas initiates the cardiorespiratory response by promoting membrane depolarization of chemoreceptive NECs (Olson et al., 2008; Porteus et al., 2014). This is also supported by the identification of CBS and CSE in the gills of rainbow trout and zebrafish whose inhibition, or gene knockdown (in zebrafish larvae), abolishes or attenuates the hyperventilatory response to hypoxia (Porteus et al., 2014). In addition, under normoxia, Na_2_S (H_2_S donor) induces membrane depolarization of trout branchial NECs (Olson et al., 2008), and increases intracellular Ca^2+^ in zebrafish (Porteus et al., 2014). Also for this gasotransmitter, the mechanism appears similar to that described in mammals.

## Cardiac nitrosative signals

### Generation of NO and its metabolites

In almost all animal tissues, NO is generated by the family of NOS isoenzymes [i.e., the constitutive endothelial (eNOS) and neuronal (nNOS), and the inducible (iNOS), isoforms], which convert L-arginine into L-citrulline and NO, in the presence of O_2_ and NADPH as essential cofactors. This reaction, because of the obligatory requirement for molecular O_2_, is vulnerable to hypoxia (Moncada and Higgs, 1993; Bryan, 2006; Lundberg et al., 2008).

NOS enzymes are the products of different genes, and show different localization, regulation, catalytic properties and inhibitor sensitivity (Pautz et al., 2010; Imbrogno et al., 2011). The constitutive NOSs produce, in the steady-state, nanomolar concentrations of NO. In contrast iNOS, if induced by immunologic and inflammatory stimuli, generates micromolar cytotoxic amounts of the gas (Vallance et al., 2000).

NO exerts its physiological effects by reversible binding and/or reacting with hemes, thiols or amines, forming iron-nitrosyl (FeNO), S-nitroso (SNO) and N-nitroso (NNO) compounds (Hill et al., 2010). NO has a very short half-life. It is rapidly metabolized to nitrite in reaction with O_2_ (Lundberg et al., 2008), and is inactivated by oxidation to nitrate in reaction with oxygenated hemoglobin (Hb) and myoglobin (Mb). NO can also react with O_2_, yielding peroxynitrite (ONOO^−^) (Ronson et al., 1999), and this depletes the bioactivity of the gas (Guzik et al., 2002). ONOO^−^ itself is not only a signaling molecule, but also a highly reactive species (Pacher et al., 2007), being able to form additional types of reactive nitrogen species, including nitrogen dioxide (NO_2_) and dinitrogen trioxide (N_2_O_3_). All reactive nitrogen species are responsible for protein post-translational modifications because of either ability to induce either S-nitrosation [the formation of a covalent bond between an NO^+^ equivalent and a nucleophilic center (amine or thiol)], or S-nitrosylation [the addition of NO without changing the formal charge of the substrate (metal center or radical species)] (Heinrich et al., 2013). Often, S-nitrosation and S-nitrosylation are used interchangeably to refer to the same substrate modification, i.e., the process leading to S-nitrosothiols (SNO) formation within proteins (for specific chemical terminology, see Heinrich et al., 2013). Likely, an uncontrolled nitrosation/nitrosylation of cysteine residues may induce nitrosative stress, with important effects on proteins activity, stability, conformation and/or ability to interact with other molecules (Foster et al., 2009).

In the presence of a reduced O_2_ availability, when the conversion of L-arginine in L-citrulline and NO is compromised, nitrite can be reduced back to NO, providing an alternative pathway for gas generation (Gladwin et al., 2005; Lundberg et al., 2009; van Faassen et al., 2009). This NO regeneration occurs through acidic disproportionation (Zweier et al., 1999) and enzymatic reduction via xanthine oxidoreductase (Millar et al., 1998), mitochondrial enzymes (Kozlov et al., 1999; Castello et al., 2006), or deoxygenated Hb (Cosby et al., 2003; Nagababu et al., 2003), Mb (Shiva et al., 2007a) and neuroglobin (Petersen et al., 2008). Under anoxia, also eNOS is capable of reducing nitrite to NO (Gautier et al., 2006). Thus, in the presence of low O_2_, NO production is gradually taken over by nitrite reduction, nitrite functioning as a pool of NO availability (Lundberg et al., 2008; van Faassen et al., 2009; Angelone et al., 2012).

**Figure 3:**
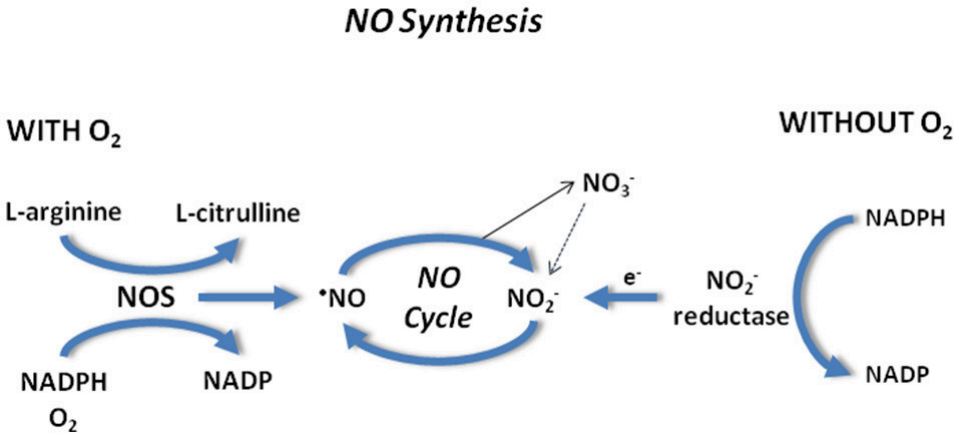
NO synthesis in the presence **(Left)** and absence **(Right)** of O_2_. Note NOS and nitrite reductase components involved in the NO formation.

Also nitrate contributes to NO homeostasis since it can be slowly reduced to nitrite by the ubiquitous enzyme xanthine oxidoreductase (Jansson et al., 2008). The nitrate-nitrite-NO pathway may be considered complementary to the classical L-arginine-NOS pathway. All these pathways partly work in parallel, but when O_2_ availability is reduced and NOS activity is decreased, nitrite reduction to NO becomes more pronounced. Thus, according to the general concept of the NO cycle in mammals, first proposed by Reutov (Reutov, 2002), ${\text{NO}}_{2}^{-}$ and ${\text{NO}}_{3}^{-}$ ions are formed as a result of non-enzymatic/enzymatic NO oxidation: L-Arg => NO =>${\text{NO}}_{2}^{-}$/${\text{NO}}_{3}^{-}$; the reduction of ${\text{NO}}_{2}^{-}$ ions to NO: ${\text{NO}}_{2}^{-}$ + e- => NO takes place through the nitrite reductase reaction (reviewed in Tota et al., 2010). The NOS and nitrite-reductase component of the NO cycle are schematically reported in Figure 3.

### The cardiac NO signaling in the response to hypoxia

#### Mammals

In mammals, the cardiac physiological role of NO in relation to O_2_ availability is well established. A hypoxia-dependent increase of NO helps mammalian myocardial cells to limit cardiac injury caused by low O_2_ supply. For example NO, by down-regulating O_2_ consumption rate, both competitively and uncompetitively inhibits O_2_ binding to mitochondrial cytochrome c oxidase (Mason et al., 2006; Erusalimsky and Moncada, 2007; Cooper et al., 2008). NO inhibition of cellular respiration mainly occurs at low O_2_ tensions; thus, particularly under hypoxia, NO may protect cellular functions by extending O_2_ availability (Hagen et al., 2003; Misfeldt et al., 2009). In oxygenated heart muscle from guinea pig, NO has been found to increase metabolic efficiency, determined by the coupling between myocardial O_2_ consumption and cardiac performance and the coupling between myocardial O_2_ consumption and ATP synthesis (Shen et al., 2001).

Of note, in the presence of ischemia, the NO-dependent suppression of the electron-transport chain, by reducing mitochondrial energy production, limits the cardiac damages induced by ischemia/reperfusion (IR) (Shiva et al., 2007b).

#### Fish

In fish, the role of NO as a major organizer of complex cardiac transduction signals has been widely assessed, and to date many data are available about its wide cardiac repertoire of actions (see for example Garofalo et al., 2009a, 2012; Imbrogno et al., 2010, 2013, 2017, 2018; Filice et al., 2017; Imbrogno and Cerra, 2017). Very recent studies extended the large variety of NO functions in fish to the mechanisms which allow to maintain the cardiovascular status and control the response to low O_2_. It is well established in fish that, if NOS activity is compromised by limited O_2_, an increased NOS expression or, alternatively, a nitrite reduction to NO, stabilize NO levels, and this contributes to protect the hypoxic myocardium (Hansen and Jensen, 2010; Sandvik et al., 2012; Imbrogno et al., 2014). A similar NOS enhanced expression can be also observed in the vasculature of the trout in response to hypoxia (McNeill and Perry, 2006).

Experimental evidences indicating the NO involvement in cardiac homeostasis of teleosts fish under hypoxic/anoxic conditions mainly derive from studies on the goldfish *C. auratus*, a champion of hypoxia tolerance. In the goldfish heart, NO inhibits mitochondrial respiration without affecting contractility (Pedersen et al., 2010). This increases myocardial efficiency (i.e., the force generated per O_2_ consumed), thus importantly contributing to maintain fish myocardial function in the presence of hypoxia or anoxia (Stecyk et al., 2004). In line with these observations, Imbrogno et al. (2014) showed that, during acute hypoxia, the goldfish heart enhances its basal performance, as well as the sensitivity to heterometric (i.e., Frank-Starling) regulation. This has been considered an important mechanism for maintaining functional and metabolic interactions between organs and tissues, required for the hypoxia tolerance of the organism. Interestingly, in the goldfish, exposure to hypoxia is accompanied by an increased myocardial NOS expression, pointing to NO generation as a crucial step for adjusting the cardiac function of the goldfish during hypoxic challenges (Imbrogno et al., 2014).

It has been also reported that the hypoxia-induced increase in NO production in goldfish heart could activate sarcolemmal K_ATP_ channels, a response that may enhance tolerance of hypoxia in this species (Cameron et al., 2003). This mechanism represents a point of convergence with the mammalian preconditioning protection of ischemic myocardium in which the opening of ATP-sensitive K^+^ channels is a crucial event (Noma, 1983).

Of note, in the hypoxic goldfish heart, the increased NOS expression is accompanied by an enhanced expression of HIF-1α. Such hypoxia-dependent cross-talk between NOS and HIF-1α represents an evolutionary conserved trait of the vertebrate response to low O_2_. In fact, in the mammalian heart, under hypoxic stress (i.e., during ischemia), HIF-1α activates a number of critical genes (Hochachka and Lutz, 2001; Liu and Simon, 2004; Semenza, 2007), including NOS (Jugdutt, 2002), and this contributes to cell survival. At the same time, high NO concentrations (>1 μM) stabilize HIF-1α (Mateo et al., 2003), thus increasing the dimeric form of the protein that, via binding to HIF responsive elements (HREs), promotes NOS expression (Mateo et al., 2003). Interestingly, in hypoxia-resistant fish, as in the case of the crucian carp, HIF-1α stabilization occurs even under normoxic conditions, suggesting a high basal expression of hypoxic-regulated genes, including NOS (Sollid et al., 2006).

As illustrated above, under hypoxic conditions, a significant source of NO is represented by nitrite. Compared to terrestrial animals, fish are exposed to an additional direct uptake of exogenous nitrite from the environmental water across the respiratory surfaces (Jensen, 2009). This external nitrite supply is an important source for the internal NO generation during severe hypoxia. An example is the crucian carp that, when exposed to deep hypoxia, takes up ambient nitrite across the gills and directs it to tissues, including the heart (Hansen et al., 2016).

In hypoxia-tolerant fish, such as the goldfish and the crucian carp, basal plasma nitrite levels are around 0.75–1.75 μM. These values are higher than those observed not only in mammals, but also in fish that are hypoxia intolerant (e.g., flounder, eelpout, oyster toadfish, brown trout) (about 0.2 μM) (Jensen, 2009; Hansen and Jensen, 2010; Sandvik et al., 2012). The reason for these high nitrite plasma levels in hypoxia-tolerant species is unclear. Presumably, this depends on the high overall NOS activity/expression under normoxia (Kleinbongard et al., 2003). Of note, fish living in nitrite-contaminated environments, have significantly high plasma nitrite, concentrations reaching the millimolar range (Bath and Eddy, 1980). At high concentrations, nitrite is toxic and can influence ion, respiratory and circulatory homeostasis (Jensen, 2009). As shown in the zebrafish, exposure to high nitrite is accompanied by very high levels of HbNO, a biomarker of NO generation from nitrate (Jensen, 2007). The consequent high nitrite-derived NO could perturb physiological processes, and may induce tissues nitrosative stress, resulting in high levels of S-nitrosylated proteins and cell damage (Jensen, 2009). For these reasons, fish need to balance the advantages of a rich ambient pool of nitrite for internal NO production with the potentially dangerous effects of nitrite-polluted habitats (Jensen and Hansen, 2011). At the same time, the possibility to maintain internal nitrite levels is particularly important for securing a source for NO production during hypoxia and anoxia, where NOS enzymes are unable to produce NO. At this purpose, the goldfish and the crucian carp possess an intrinsic ability to increase intracellular nitrite concentration and nitrosylated compounds during deep hypoxia and anoxia in tissues with high myoglobin and mitochondria content, such as the heart (Sandvik et al., 2012; Jensen et al., 2014; Hansen et al., 2016). This occurs at the expenses of extracellular nitrite. The extracellular-intracellular transfer of nitrite is facilitated by nitrite binding to intracellular proteins that, by keeping low the cytosolic concentration of free nitrite, allows inward diffusion (Hansen and Jensen, 2010). As shown in the crucian carp, anoxia increases tissue nitrite in the heart, but not in the white muscle. This is indicative of a role for Mb nitrite reductase activity, which is present at high levels in the heart and in the red musculature (Jensen et al., 2014).

Although no clear evidence is available, in fish, mitochondria may play a role in elevating intracellular nitrite during hypoxia and anoxia. Indeed, the cytoprotective effects of nitrite under low O_2_ are largely directed at the mitochondria (Walters et al., 2012; de Lima Portella et al., 2015). In mammalian mitochondria, nitrite S-nitrosates complex I, attenuating ROS generation during early reperfusion (Dezfulian et al., 2009; Chouchani et al., 2013), and nitrosylates complex IV, which inhibits O_2_ consumption rates (Hendgen-Cotta et al., 2008). Unlike mammals, in hypoxia-tolerant ectotherms, as presumably in all ectotherms, reoxygenation does not affect mitochondria, which maintain their capacity to produce energy. For example, reoxygenation does not result in mitochondria Ca^2+^ overload and/or in reversing ATP-synthase into an ATPase (Galli and Richards, 2014 for references). At the same time, mitochondrial proton leak is kept low (Galli and Richards, 2014 for references). With respect to hypoxia-intolerant, in hypoxia-tolerant species, mitochondria respiration appears more resistant to hypoxic stresses. For example, among elasmobranchs, cardiac mitochondria from the hypoxia-tolerant epaulet shark (*Hemiscyllium ocellatum*) produce less reactive oxidative species than the hypoxia sensitive shovelnose ray (*Aptychotrema rostrata*) (Hickey et al., 2012). For this purpose, the dynamic organization of respiratory chain complexes and ATP synthase (Cogliati et al., 2016) results crucial for mitochondrial respiration under hypoxia.

Unique natural animal models to analyse the role of NO, nitrite and Mb in the response to hypoxia are Antarctic teleosts. Some of them are example of disaptation, being naturally deprived of Hb, and/or of cardiac Mb, as in the case of the icefish *Chionodraco hamatus* (Hb^−^/Mb^+^), *Chenocephalus aceratus* (Hb^−^/Mb^−^), and their red-blooded counterparts *Trematomus bernacchii* (Hb^+^/Mb^+^) (Garofalo et al., 2009b). This condition makes these teleosts well suited for comparatively studying evolutionary and mechanistic aspects of the NO-nitrite system in relation to cardiac homeostasis and adaptation, including the response to varying O_2_ levels (see for references Garofalo et al., 2009b). This aspect is crucial in the stably ice Antarctic waters, which are highly oxygenated but, at the same time, exposed to changes in O_2_ content. At the moment, no data are available in Antarctic teleost on the putative role of the NO/nitrite equilibrium in relation to the response to low O_2_. In the heart of *C. hamatus* and *T. bernacchii*, under normoxia, nitrite influences cardiac performance by inducing a concentration-dependent increase of contractility (Garofalo et al., 2015). In the Antarctic Hb- and Mb-less (Hb^−^/Mb^−^) icefish *C. aceratus*, intracardiac NOS expression is lower than in its Mb^+^ counterpart, the Hb^−^/Mb^+^ *C. hamatus* (Amelio et al., 2006). Compared to the Mb expressing *C. hamatus*, in *C. aceratus* the heart is more sensitive to NOS stimulation by L-arginine (Cerra et al., 2009). Since the nitrite reductase activity of cardiac Mb is absent and NOS is poorly expressed, other mechanisms have been proposed to contribute to local NO production. For example, in the absence of the Mb-mediated scavenging effect, NO half-life is increased; the consequent larger availability of free NO may compensate for the reduced NOS expression. Contrarily, in *C. hamatus*, cardiac Mb may contribute to local NO generation and this maintains the nitrergic homeostasis. Interestingly, in *C. hamatus*, very low concentrations of exogenous nitrite (0.1 μmol/l) increase cardiac contractility, an effect similar to that elicited by NO (Cerra et al., 2009). At the same time, as in mammals (Vanin et al., 2007), the largely expressed NOS equipment might represent a rich source of NO from nitrite. Of note, in Hb^−^/Mb^−^ icefish, the high NO levels occurring in the absence of both respiratory proteins correlate with major cardiovascular and subcellular compensations, including mitochondrial enlargement within myocardiocytes (Urschel and O'Brien, 2008). This contributes to myocardial oxidative equilibrium and hence to heart protection under hypoxia.

## Conclusions

This review emphasizes the amazing flexibility of teleost fish in relation to their peculiar ability to cope with low O_2_. Teleosts possess complex equipments for sensing O_2_ that activate intricate downstream molecular signal-transduction networks crucial to balance tissue O_2_ supply and demand. Although much still remains to be investigated, the available information indicate the critical role played by NO and its metabolites in the physiological strategies that in teleost allow cardiac adaptive responses to O_2_ limitation, also contributing to better understand the extraordinary morphofunctional plasticity and adaptation that determined their evolutionary success.

## Author contributions

AG and FG participated in drafting, writing, and editing the manuscript. MCC and SI participated in writing and editing the manuscript. All Authors approved it for publication.

### Conflict of interest statement

The authors declare that the research was conducted in the absence of any commercial or financial relationships that could be construed as a potential conflict of interest.
